# A review and bibliometric analysis of global research on proton radiotherapy

**DOI:** 10.1097/MD.0000000000038089

**Published:** 2024-05-10

**Authors:** Ge Song, Zhi Zheng, Yingming Zhu, Yaoting Wang, Song Xue

**Affiliations:** a Department of Critical Care Medicine, Shandong Provincial Maternal and Child Health Care Hospital Affiliated to Qingdao University, Jinan, China; b Department of Stomatology, Shandong Provincial Maternal and Child Health Care Hospital Affiliated to Qingdao University, Jinan, China; c Department of Radiation Oncology, National Cancer Center/National Clinical Research Center for Cancer/Cancer Hospital, Chinese Academy of Medical Sciences and Peking Union Medical College, Beijing, China; d Department of Oncology, Dongying People’s Hospital, Dongying, China; e Experimental Center, Shandong University of Traditional Chinese Medicine, Jinan, China.

**Keywords:** bibliometrics, cancer, CiteSpace, proton beam therapy, radiation, visualization analysis, visualization research

## Abstract

Proton beam therapy (PBT) has great advantages as tumor radiotherapy and is progressively becoming a more prevalent choice for individuals undergoing radiation therapy. The objective of this review is to pinpoint collaborative efforts among countries and institutions, while also exploring the hot topics and future outlook in the field of PBT. Data from publications were downloaded from the Web of Science Core Collection. CiteSpace and Excel 2016 were used to conduct the bibliometric and knowledge map analysis. A total of 6516 publications were identified, with the total number of articles steadily increasing and the United States being the most productive country. Harvard University took the lead in contributing the highest number of publications. Paganetti Harald published the most articles and had the most cocitations. PHYS MED BIOL published the greatest number of PBT-related articles, while INT J RADIAT ONCOL received the most citations. Paganetti Harald, 2012, PHYS MED BIOL can be classified as classic literature due to its high citation rate. We believe that research on technology development, dose calculation and relative biological effectiveness were the knowledge bases in this field. Future research hotspots may include clinical trials, flash radiotherapy, and immunotherapy.

## 1. Introduction

Radiation therapy is one of the treatment strategies employed against malignant tumor cells, and it is estimated that approximately 50% of cancer patients undergo radiotherapy during their treatment course.^[1,2]^ Traditional radiotherapy employs photon beams for administering a concentrated dose of radiation to the tumor area with the aim of eliminating cancer cells. Nevertheless, the physical properties of photons pose challenges in safely escalating doses while adhering to constraints on normal tissue tolerance.^[3]^

Proton beam therapy (PBT), a type of cutting-edge therapeutic radiation, utilizes charged particles with physical properties that inherently reduce the amount of excess radiation delivered to patients when compared with photon-based radiotherapy. Protons exhibit a Bragg peak in their dose distribution, indicating maximal dose deposition at a finite tissue depth followed by a sharp dose falloff with no exit dose.^[4]^ As such, PBT allows for an increase in the radiation dosage to reach the tumor while concurrently minimizing toxicity in normal tissues, thereby expanding the therapeutic window for individuals with cancer. According to the Particle Therapy Cooperative Group (PTCOG) website (http://www.ptcog.ch), as of December 2023, there were approximately 113 operational proton therapy facilities worldwide and 32 more under construction. As PBT has become widely adopted in cancer treatment, related studies on PBT for tumors are accumulating. Therefore, retrospective analysis of published PBT articles is imperative and can help researchers determine the current problems to provide some suggestions for follow-up development in this field.

Bibliometric analysis is a statistical method for evaluating the literature and exploring trends in a research field through quantitative analysis of related scientific literature.^[5]^ CiteSpace is an effective tool for bibliometric analysis that can scan a vast number of articles to qualitatively and quantitatively evaluate research in nations, institutions, and specific topic areas. This tool aids scholars in understanding the developmental characteristics of a field and provides valuable insights to guide future research endeavors. In the present study, for the first time, we used this software to qualitatively evaluate the literature on PBT to analyze the contributions of countries/regions, institutions, journals, and authors; detect collaboration among authors, nations, and institutions.; and explore hotspots and future research trends in this area.

## 2. Methods

### 2.1. Data source and search strategy

The Web of Science Core Collection database was reviewed to obtain relevant literature from inception to December 31, 2023. The search strategy was defined as follows: TS (topic searches) = (“proton beam therapy” OR “proton therapy” OR “pencil beam proton therapy” OR “pencil beam scanning” OR “intensity modulated proton beam therapy” OR “proton treatment*” OR “proton radiotherapy”). Document types were bound to articles. All searches were conducted on the same day to mitigate any bias resulting from daily database updates.

### 2.2. Data analysis

Microsoft Office Excel 2016 (Redmond, WA) was used to analyze the trend of the number of annual publications. The impact factors (IFs) for all publications were recorded using data from the Journal Citation Report (2023).

CiteSpace (version 6.3.R1) was used for visualizing cocitation and co-occurrence network. In the atlas, nodes represent analytical characteristics (such as authors, institutions, countries, journals, cocited authors, and references), and links between nodes usually represent cooperative or cocitation relationships. The colors of the nodes and lines change depending on the year. The size of rings on nodes indicates the number of publications or frequency for each node. Purple rings surrounding circles indicate the centrality of nodes, and nodes with a larger centrality are often seen as key points in the network.^[6]^

We also performed cluster analysis for references and burst detection for keywords. The keyword terms and a log-likelihood ratio weighting algorithm were used to mark the clusters.^[7]^ The quality of the cluster networks was evaluated using modularity (Q) and the silhouette (S) method. A cluster structure is considered significant when Q is greater than 0.3. A cluster is considered reasonable when S is greater than 0.5 and convincing when S is greater than 0.7.^[8]^ Burst detection was used to identify current and future areas of focus. When the period in burst detection is red, it means that the element is undergoing a citation burst during the period, which may indicate a potential trend in the field.

## 3. Results

### 3.1. Description and trends of publications

**Figure 1.**
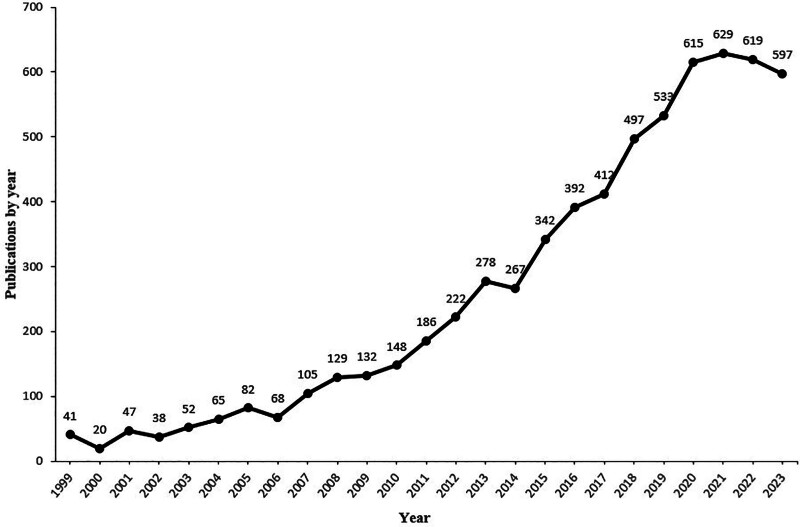
Distribution of publications by year.

We created a line chart illustrating the growth trend of proton radiotherapy research based on the annual number of publications. Publications about proton radiotherapy are increasing annually, with a peak in 2021 (Fig. 1). Prior to 2012, the annual publications were fewer than 200. After 2014, the number of articles increased rapidly, and over 400 articles were published in 2017, 500 in 2019, and 600 in 2020. As of December 31, 2023, a total of 597 pertinent papers had been published in this year.

### 3.2. Countries/regions and institutions

**Figure 2.**
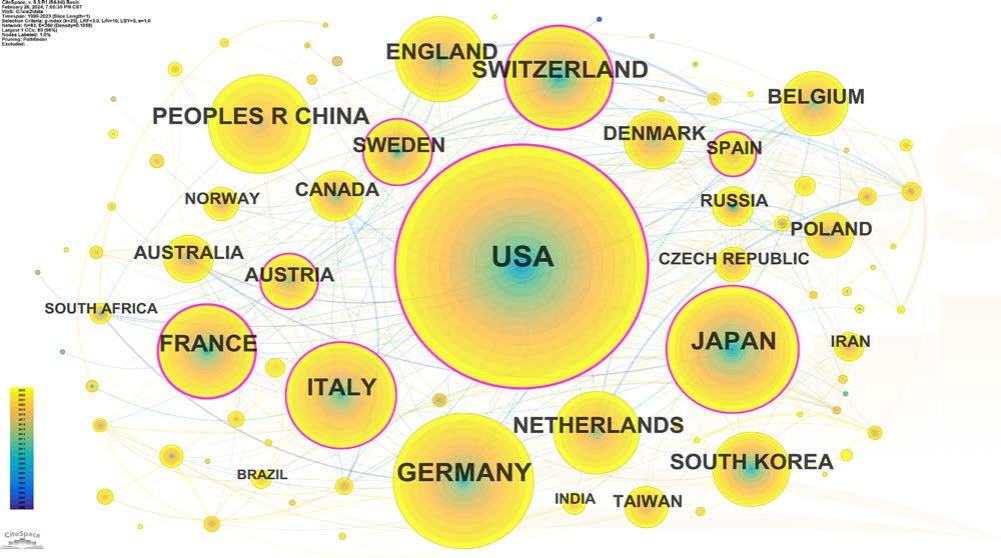
A visualization of the country collaboration network.

All the publications were distributed among 83 countries/regions. The country with the largest output of publications was the United States (n = 2565, accounting for 40.3% of the total), followed by Germany (n = 887, 13.9%), Japan (n = 750, 11.8%), Italy (n = 559, 8.8%) and Switzerland (n = 519, 8.1%) (Table 1). Among the top 10 countries, the United States, France and Switzerland had higher centrality, 0.37, 0.24, and 0.20, respectively. This indicated that these nations played a pivotal role in facilitating cooperation among countries (Fig. 2).

**Table 1:** The top 10 countries and institutes in terms of publication numbers in PBT research.

Rank	Country/region	Count	Percentage (%)	Centrality	Institution	Count	Centrality
1	USA	2565	40.38	0.37	Harvard University (USA)	570	0.04
2	Germany	887	13.96	0.10	University of Texas System (USA)	556	0.08
3	Japan	750	11.81	0.11	Massachusetts General Hospital (USA)	525	0.14
4	Italy	559	8.80	0.14	MD Anderson Cancer Center (USA)	517	0.09
5	Switzerland	519	8.17	0.20	Helmholtz Association (Germany)	502	0.15
6	France	442	6.96	0.24	Harvard Medical School (USA)	402	0.17
7	China	434	6.83	0.01	Swiss Federal Institutes of Technology Domain (Switzerland)	383	0.10
8	England	362	5.70	0.07	Paul Scherrer Institute (Switzerland)	366	0.21
9	Netherlands	338	5.32	0.04	German Cancer Research Center (Germany)	330	0.10
10	South Korea	282	4.44	0.05	UDICE-French Research Universities (France)	295	0.16

PBT = proton beam therapy.

Overall, 4295 institutions contributed to this field. The most productive institution was Harvard University, with 570 publications, followed by the University of Texas System (556), the Massachusetts General Hospital (525), and the MD Anderson Cancer Center (517) (Table 1). The 4 most prolific institutions were all in the US. In addition, the institution with the highest centrality (0.21) was the Paul Scherrer Institute, indicating that it plays an important role in this research field among top institutions. As depicted in Figure S1, Supplemental Digital Content, http://links.lww.com/MD/M469, an intricate collaborative relationship was evident among the major institutions.

### 3.3. Authors and cocited authors

**Figure 3.**
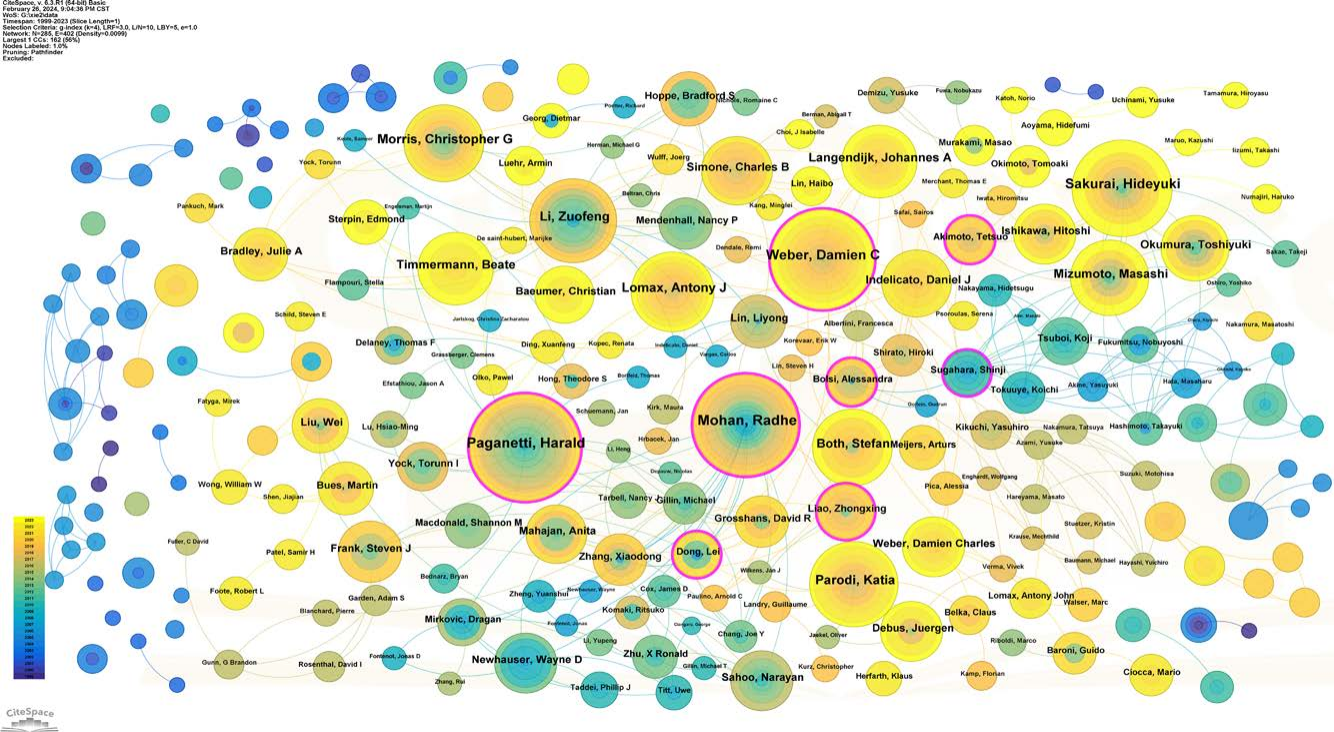
A visualization of the author collaboration network.

A total of 21,242 authors contributed to this field of research. Paganetti Harald is identified as the most productive author, with 138 publications, and Mohan Radhe ranks second, followed by Sakurai Hideyuki, Parodi Katia, and Weber Damien C (Table 2). At least 68 papers were contributed by each of the top 10 authors. A network view map illustrated the collaborations among the active authors (Fig. 3).

**Table 2:** The top 10 authors and cocited authors in PBT research.

Rank	Authors	Count	Cocited author	Citations
1	Paganetti Harald	138	Paganetti Harald	2403
2	Mohan Radhe	110	Lomax Antony J	983
3	Sakurai Hideyuki	105	Schneider Uwe	976
4	Parodi Katia	94	Parodi Katia	908
5	Weber Damien C	92	Pedroni Eros	690
6	Li Zuofeng	78	Agostinelli S	558
7	Mizumoto Masashi	76	Liu Wei	553
8	Lomax Antony J	74	Unkelbach Jan	527
9	Morris Christopher G	70	Chang Joe Y	524
10	Okumura toshiyuki	68	Newhauser, Wayne D.	475

PBT = proton beam therapy.

Table 2 lists the top 10 cocited authors. Among the top 10 cocited authors, Paganetti Harald ranked first, with 2403 citations, followed by Lomax Antony J (983 citations), Schneider Uwe (976 citations) and Parodi Katia (908 citations); the remaining authors had fewer than 475 citations.

### 3.4. Journals and cocited journals

All 6516 papers were published in 628 journals. Among the top 10 journals with the most publications (Table 3), PHYSICS IN MEDICINE AND BIOLOGY published the most papers (818 papers), followed by MEDICAL PHYSICS (670 papers), INTERNATIONAL JOURNAL OF RADIATION ONCOLOGY BIOLOGY PHYSICS (482 papers), RADIOTHERAPY AND ONCOLOGY (313 papers), and JOURNAL OF APPLIED CLINICAL MEDICAL PHYSICS (185 papers).

**Table 3:** The top 10 journals and cocited journals in PBT research.

Rank	Journal	N	IF (2023)	Cocited journal	Cocitation	IF (2023)
1	PHYS MED BIOL	818	3.5	INT J RADIAT ONCOL	27,900	7.0
2	MED PHYS	670	3.8	PHYS MED BIOL	24,554	3.5
3	INT J RADIAT ONCOL	482	7.0	MED PHYS	17,886	3.8
4	RADIOTHER ONCOL	313	5.7	RADIOTHER ONCOL	10,185	5.7
5	J APPL CLIN MED PHYS	185	2.1	J CLIN ONCOL	4813	45.3
6	PHYS MEDICA	182	3.4	NUCL INSTRUM METH A	3669	1.3
7	ACTA ONCOL	165	3.1	ACTA ONCOL	2914	3.1
8	RADIAT ONCOL	160	3.6	RADIAT ONCOL	2538	3.6
9	NUCL INSTRUM METH A	159	1.3	CANCER-AM CANCER SOC	1967	6.2
10	CANCERS	122	5.2	RADIAT RES	1960	2.6

PBT = proton beam therapy.

Table 3 displays the top 10 most frequently cited journals. INTERNATIONAL JOURNAL OF RADIATION ONCOLOGY BIOLOGY PHYSICS is the most common journal, with 27,900 citations. This journal has had a profound impact on related research in this field, followed by PHYSICS IN MEDICINE AND BIOLOGY (24,554 citations) and MEDICAL PHYSICS (17,886).

### 3.5. Cocited references and cluster analysis

We present the 10 most cited references in Table S1, Supplemental Digital Content, http://links.lww.com/MD/M470. Among the top 10 cited references, an article by Paganetti H et al was the most common, as this work analyzed various factors affecting range uncertainty (RU) and summarized the role of Monte Carlo simulations when aiming at reducing RU in proton therapy.^[9]^ The reference conducted by Agostinelli S et al^[10]^ (532 cocitations) had the second highest number of cocitations, followed by the articles performed by Paganetti H et al^[11]^ (324 cocitations); the remaining 7 references were cocited between 200 and 300 times.

**Figure 4.**
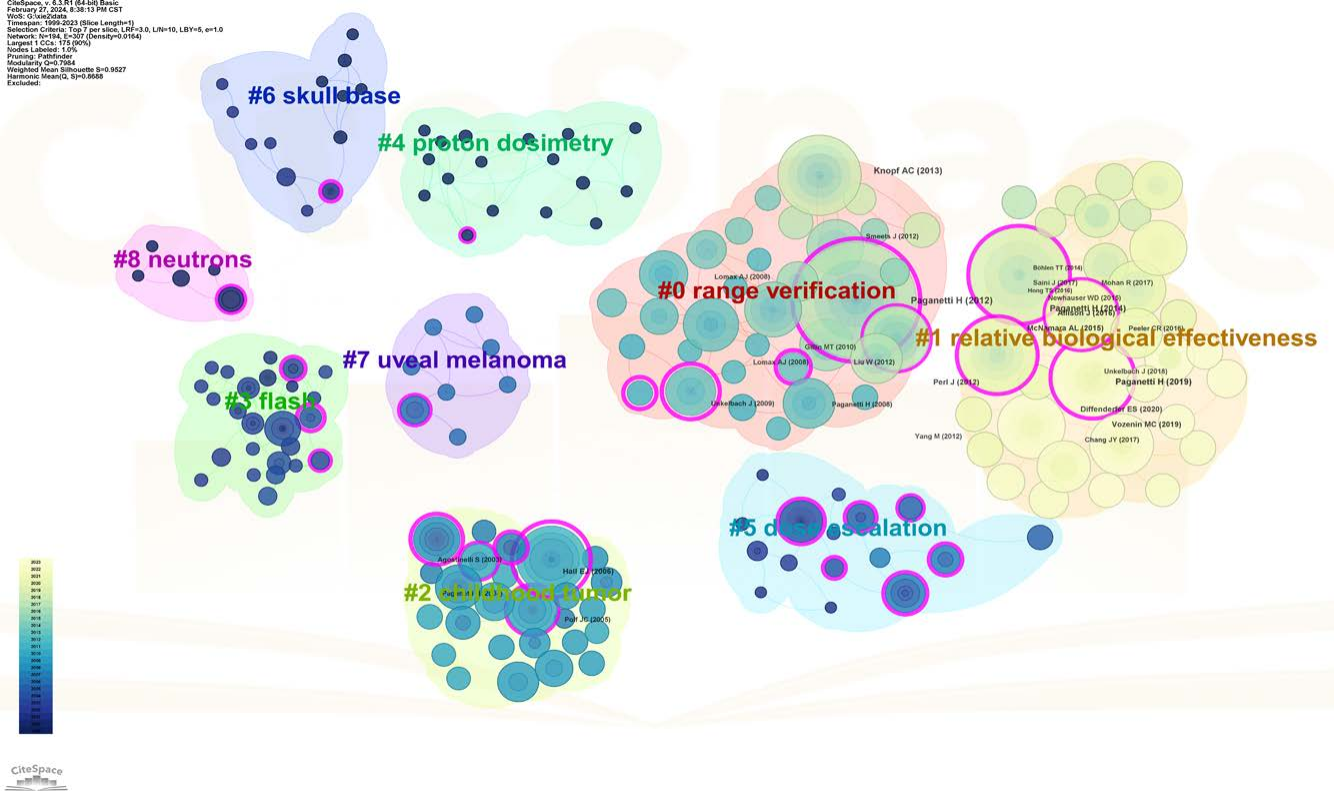
Cocitation relationships between references. The figure shows 9 color blocks representing 9 clusters, each composed of articles on the same topic.

By clustering, the set of all cited references was divided into clusters with different labels. The modularity Q (0.7954) and mean silhouette (0.9527) values were greater than 0.3 and 0.7, respectively. In total, 9 clusters were identified (Fig. 4). The first cluster label on the knowledge map was “#0 range verification,” and the second cluster label was “#1 relative biological effectiveness.” The remaining 7 clusters were named “#2 childhood tumor,” “#3 flash,” “#4 proton dosimetry,” “#5 dose escalation,” “#6 skull base,” “#7 uveal melanoma,” and “#8 neutrons.” These clusters indicate a major portion of the PBT research.

### 3.6. Keyword co-occurrence and burst analysis

Analyzing the co-occurrence of keywords can unveil research focal points, with frequently occurring keywords often indicating the primary research direction within the field. Since some keywords were the same, we merged these words. Table 4 shows that the top 20 keywords appear more than 200 times. The most frequently occurring keyword was proton therapy (n = 3137), followed by radiotherapy (n = 2779), therapy (n = 781), cancer (n = 756), irradiation (n = 568), intensity-modulated radiotherapy (n = 551) and uncertainty (n = 473). These keywords represent the hotspots of PBT research.

**Table 4:** The top 20 keywords in PBT research.

Rank	Keyword	Count	Rank	Keyword	Count
1	proton therapy	3137	11	relative biological effectiveness	348
2	radiotherapy	2779	12	dosimetry	344
3	therapy	781	13	optimization	326
4	cancer	756	14	risk	315
5	irradiation	568	15	head	311
6	intensity-modulated radiotherapy	551	16	outcomes	306
7	uncertainty	473	17	proton	299
8	monte carlo	420	18	survival	269
9	radiation	398	19	photon	261
10	system	367	20	chemotherapy	248

PBT = proton beam therapy.

**Figure 5.**
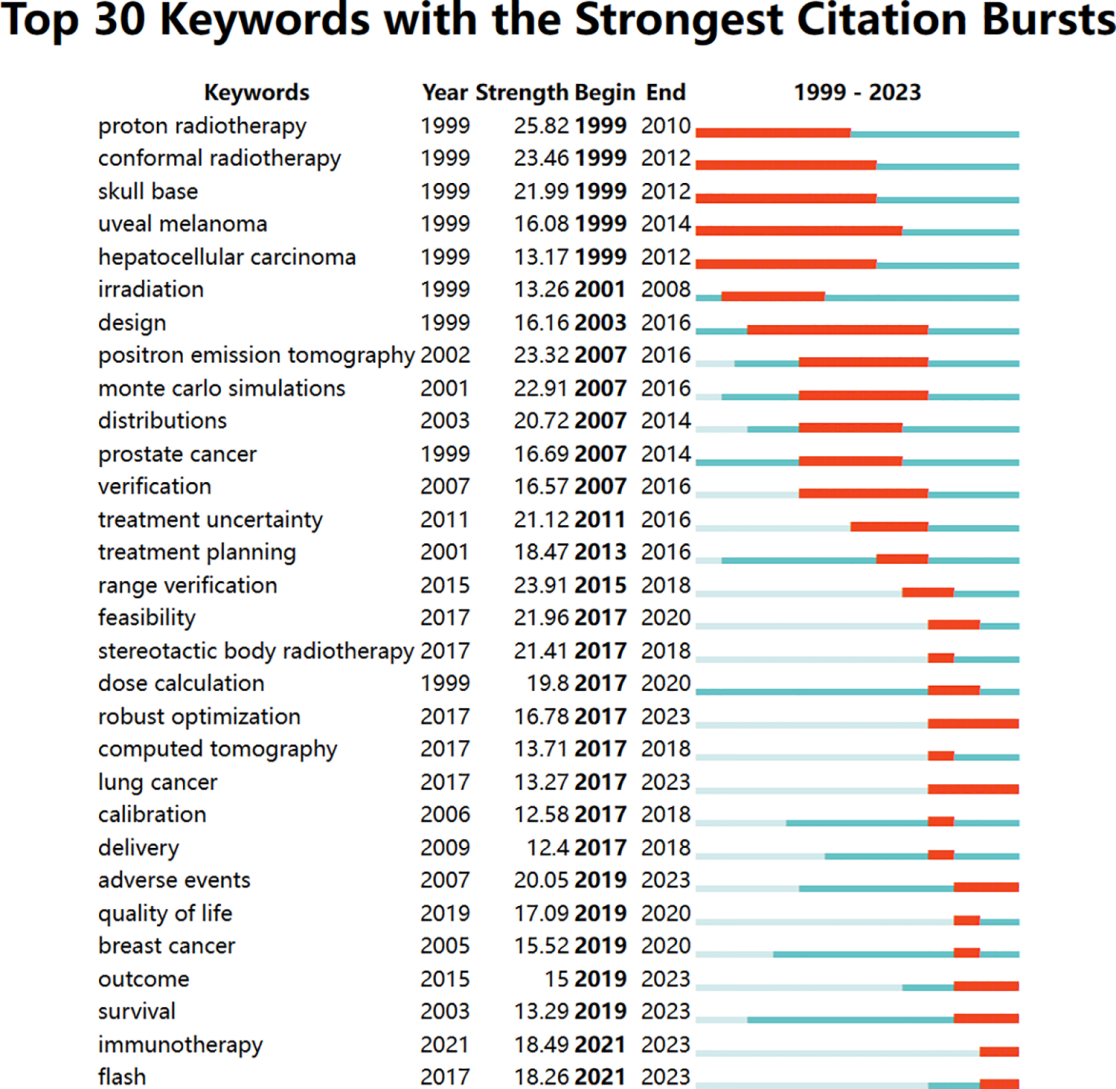
Detection of the top 30 keywords for citation bursts.

For a more in-depth exploration of evolving research hotspots over time, we employed the built-in burst detection algorithm in CiteSpace. We detected 30 keywords that had citation bursts (Fig. 5). Of the 30 keywords with citation bursts, “proton radiotherapy” had the highest burst strength (25.82). The keywords “outcome,” “survival,” “immunotherapy,” and “flash” were associated with recent citation bursts, and the term “uveal melanoma” was used to indicate the longest duration of disease occurrence from 1999 to 2014.

## 4. Discussion

### 4.1. General information

The therapeutic capabilities of protons were initially acknowledged in 1946, as documented in a report by Wilson.^[12]^ By as early as 1954, the University of California administered proton irradiation to its first patient.^[13]^ Since that time, the number of publications on PBT for tumors has increased steadily, which was consistent with the rising trend of proton therapy facilities counted on the PTCOG website. In contrast to the peak in 2021, the volume of articles in 2022 has decreased but remains at a high level, which indicates that PBT will continue to receive increased amounts of attention in the future.

Regarding regional productivity, the top 10 most prolific countries were dominated by developed countries, except for China. As the leading force, the United States has contributed a great volume of publications and has engaged in frequent collaborations with other countries. The institutional distribution was generally consistent with the country distribution. The top 10 research institutes with the most publications are all derived from developed countries, with 5 institutes from the USA, 2 institutes from Germany, 2 institutes from Switzerland, and one from France. This phenomenon can be attributed to the substantial financial support required for the research and development of technology related to PBTs. Moreover, PBT is also an expensive treatment, especially compared to conventional photon radiotherapy.

Physics In Medicine and Biology was the most productive journal in this field, with an IF of 3.5. The International Journal of Radiation Oncology • Biology • Physics has a lower volume of publications, but it has a significant influence on the field, as it is the most cited, earning a high IF of 7.9. This underscores its standing as an influential journal in the domain. In addition, we list the 10 leading contributing authors and the 10 authors with the most citations, who all have devoted themselves to conducting research in the field. Notably, Paganetti Harald was the most prolific author and was also the author with the most citations. Therefore, Paganetti Harald is considered a pioneering and influential researcher in this field.

### 4.2. Knowledge base

Cocited references are recognized as a knowledge base, and can also be regarded as a primary focus for researchers in a particular field.^[14,15]^ Based on the top 10 cocited references and clustering analysis, we identified the research topics of major interest as follows:

#### 4.2.1. Technology development

Protons are heavy, charged particles that can be stripped from hydrogen gas and accelerated to therapeutic energies (typically from 70–250 MeV) using a cyclotron or a synchrotron;^[16]^ each has advantages and disadvantages. Cyclotrons can be very compact using superconducting technology, while they draw a continuous stream of protons that can achieve a high beam intensity. The energy of the protons induced by the cyclotron is too high, so the needed lower energies are achieved by energy degraders.^[17]^ However, the energy of proton beam produced by a synchrotron is adjustable and can be tailored to meet the needs of clinical application. However, synchrotrons occupy a relatively larger footprint than cyclotrons.^[18]^

The initial accelerated proton beam is very thin and is unsuitable for treating three-dimensional tumor targets with arbitrary shapes. Scattering foils and brass apertures can be utilized to spread and shape proton beams for “passively scattered proton therapy”.^[19]^ The Intensity-modulated proton therapy represents an alternative proton beam delivery technique utilizing pencil beam scanning. This approach enables larger treatment sizes and enhances flexibility in dose-shaping efficiency and dose conformity.^[20,21]^

#### 4.2.2. Dose calculation

Due to energy fluctuations, the range at which the proton beam terminates is subject to some uncertainty, which can result in underdosing the target volume or overdosing critical structures.^[22]^ One significant source of uncertainty arises from approximations made in the methods used for dose computations within treatment planning systems.^[23,24]^ At present, commercial treatment planning systems typically compute proton dose distributions using analytical algorithms, which have fast computational speeds and can meet the needs of clinical treatment. However, this algorithm of proton dose computations involves numerous assumptions and approximations and is less sensitive to complex geometries and density variations, resulting in low accuracy in dose calculations.

The Monte Carlo algorithm has improved the accuracy of dose calculations and has been designated as the gold standard for modeling doses, but its clinical application is limited due to its slow calculation speed. As stated by Paganetti et al, Monte Carlo dose calculation takes approximately 6 hours per patient.^[25]^ To address the issue of computational speed, scientists are working on accelerated Monte Carlo methods that offer comparable accuracy to full-fledged Monte Carlo methods but are at least 100 times faster.^[26]^ In addition to accelerating Monte Carlo, Kohno et al devised a Simplified Monte Carlo (SMC) method based on a graphical processing units (GPU) and clinically implemented it in patients with head and neck, lung, or prostate cancer. In all the cases, the developed GPU-SMC led to a reduction in the computation time.^[27]^ The accuracy of GPU-based Monte Carlo tool was reported to be sufficient in most cases, with a gamma passing rate exceeding 94% for voxels within the 10% isodose line. It’s worth noting that, however, ~2% systematic overestimation of dose in the entrance region and 1% to 2% underestimation in the target was observed for prostate cancer cases.^[28]^ Therefore, accelerated Monte Carlo methods still left room for improvement regarding its accuracy and suitability for clinical uses.

#### 4.2.3. Relative biological effectiveness

Relative biological effectiveness (RBE) was defined as the ratio of the absorbed dose of a reference radiation to that of a test radiation that produced the same biological effect. The RBE of protons is assumed to have a constant value of 1.1.^[11]^ This value of RBE is established through the averaging of outcomes from numerous in vitro and in vivo experiments conducted under diverse conditions. These experiments are frequently carried out at high doses per fraction and in the middle of the spread-out Bragg peak, where the RBE remains relatively constant and close to the average value of 1.1.

However, in reality, the RBE is variable and depends on several factors, such as the energy of protons, dose per fraction, tissue and cell type, and end point.^[29]^ It may approach 1 in the entrance regions and considerably increase to 1.7 in the distal dose fall-off region of the spread-out Bragg peak.^[30,31]^ If the region of low RBE is located within the tumor volume or if the region of high RBE is in normal tissue, the expected benefits of proton therapy may be compromised, leading to unexpected recurrences or toxicities.

### 4.3. Research hotspots and emerging topics

The utilization of co-occurrence keyword and burst analyses allows for the assessment of trending research topics and the identification of emerging areas within a specific field.^[32]^ Early researchers focused more on the clinical application of PBT, especially in refractory tumors and small target volumes, including eye tumors. In addition, the physical properties of protons are thought to be of special benefit for pediatric patients requiring radiotherapy for brain tumors due to the intricate radiation sensitivity of children’s normal tissue as well as the potential for long-term survivorship.^[33]^ The keywords of interest included “monte carlo simulations,” “verification,” “RBE” and “range uncertainty,” indicating that the accuracy of dose distribution and radiobiology studies are hotspots in the field of PBT. In recent years, an increasing number of clinical trials have explored outcome issues, such as survival time, quality of life and adverse events, in cancer patients to guide the clinical development of a more rational treatment strategy.

Currently, researchers have gradually explored new beam delivery methods. FLASH radiotherapy is a method for delivering a therapeutic dose at ultrahigh dose rates exceeding 40 Gy/s, which could reduce damage to normal tissues while preserving the ability to treat tumors.

While proton-FLASH studies may not be as prevalent as those conducted with electrons or X-ray photons, numerous preclinical studies affirm the correlation between ultrahigh dose rates and normal tissue protection.^[34–36]^ For example, an in vitro study showed that FLASH irradiation could attenuate late adverse biological effects;^[37]^ another in vivo study published in 2020 showed that proton-FLASH-irradiated mice demonstrated significantly diminished levels of acute intestinal damage.^[38]^ However, it should be noted that clinical applications of proton-FLASH are in their early stages, and the mechanism underlying the effect of FLASH syndrome remains unclear.

In the era of cancer immunotherapy, the question of combining the distinct radiobiological and dosimetric properties of proton beam therapy with immunotherapy to improve patient outcomes is an area worth investigating. Preclinical studies provide evidence supporting the immunogenic potential of proton therapy, indicating that it might have broader applications in immunotherapy than photon therapy. For example, in vitro data suggest that while treatment with protons and photons results in a similar surviving fraction of melanoma cells, protons can induce long-term inhibition of cell migration. This translates to a reduced likelihood of distant tumor spread.^[39]^ An in vitro study has also shown that proton irradiation mediates calreticulin translocation to the cell surface, which increases cross-priming and sensitivity to cytotoxic T lymphocytes.^[40]^ Clinically, Su et al reviewed 29 patients with advanced hepatocellular carcinoma who received PBT and PD-1/PD-L1 (programmed cell death protein 1/programmed cell death 1 ligand 1) inhibitors during 2016 and 2019. PBT combined with PD-1/PD-L1 inhibitors was considered safe and produced no unexpected adverse events.^[41]^ In a single-arm, prospective phase II clinical trial of recurrent or metastatic head and neck squamous cell carcinoma, durvalumab plus tremelimumab combined with proton therapy was shown to have encouraging antitumor efficacy and tolerable safety profiles.^[42]^ Indeed, some clinical trials have already been ongoing or are recruiting patients, and within a few years, the role of PBT in combination with cancer immunotherapy is expected to be defined.

### 4.4. Limitations

First, this was a bibliometric study, and the utilization of CiteSpace software could not entirely substitute for systematic retrieval methods. Second, the data were retrieved from only the Web of Science Core Collection and included research articles, which may not comprehensively capture the current state of all PBT research. Third, as some authors share the same short name and certain keywords have varied expressions, despite our standardization efforts, biases may still be present.

## 5. Conclusions

To our knowledge, this is the first study in which a bibliometric analysis of PBT research was performed by using CiteSpace. The number of publications on PBTs has increased steadily, and the United States has led in this area of research. PHYS MED BIOL, MED PHYS and INT J RADIAT ONCOL are the top 3 productive journals and top 3 cocited journals. Paganetti Harald is the top author and the top cocited author with the highest influence in this field. The analysis of keywords and references revealed that technology development, dose calculations and RBE are knowledge bases and research hotspots. Proton-FLASH radiotherapy, proton therapy combined with immunotherapy, and the outcome of clinical trials may be the spotlight for future research. In conclusion, we believe that this article can offer some inspiration to researchers and clinicians engaged in relevant fields.

## Author contributions

**Conceptualization:** Song Xue.

**Data curation:** Zhi Zheng, Yingming Zhu.

**Formal analysis:** Yaoting Wang.

**Software:** Zhi Zheng.

**Supervision:** Song Xue.

**Writing – original draft:** Ge Song, Zhi Zheng.

**Writing – review & editing:** Ge Song, Song Xue.

## Supplementary Material

**Figure:**
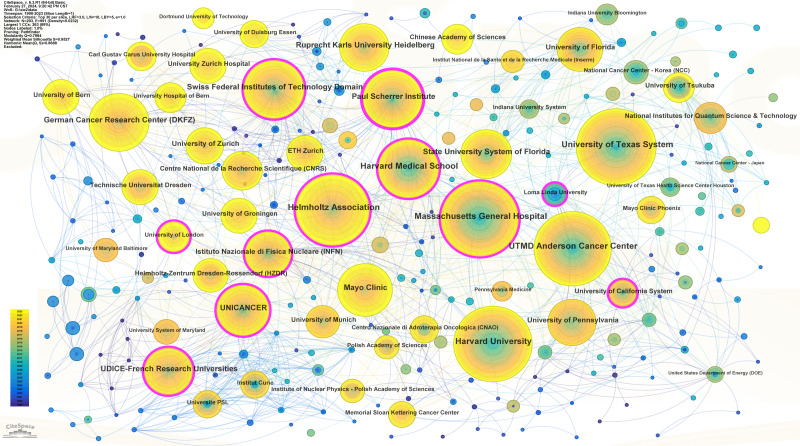




## References

[1] Harrington KJ Billingham LJ Brunner TB. Guidelines for preclinical and early phase clinical assessment of novel radiosensitisers. Br J Cancer. 2011;105:628–39. 21772330 10.1038/bjc.2011.240 PMC3188925

[2] Herrera FG Bourhis J Coukos G. Radiotherapy combination opportunities leveraging immunity for the next oncology practice. CA Cancer J Clin. 2017;67:65–85. 27570942 10.3322/caac.21358

[3] Lu Z Zheng X Ding C. Deciphering the biological effects of radiotherapy in cancer cells. Biomolecules. 2022;12:1167. 36139006 10.3390/biom12091167 PMC9496570

[4] Byun HK Han MC Yang K. Physical and biological characteristics of particle therapy for oncologists. Cancer Res Treat. 2021;53:611–20. 34139805 10.4143/crt.2021.066 PMC8291193

[5] Nicolaisen J. Bibliometrics and citation analysis: from the science citation index to cybermetrics. J Assoc Inf Sci Technol. 2010;61:205–7.

[6] Chen C Hu Z Liu S Tseng H. Emerging trends in regenerative medicine: a scientometric analysis in CiteSpace. Expert Opin Biol Ther. 2012;12:593–608. 22443895 10.1517/14712598.2012.674507

[7] Li R Liu X Yang B Qiu J. External beam radiotherapy for prostate cancer: What are the current research trends and hotspots? Cancer Med. 2021;10:772–82. 33480190 10.1002/cam4.3700 PMC7877352

[8] Zhan Q Wu C Ding H. Emerging trends in photodynamic therapy for head and neck cancer: a 10-year bibliometric analysis based on CiteSpace. Photodiagnosis Photodyn Ther. 2022;38:102860. 35429646 10.1016/j.pdpdt.2022.102860

[9] Paganetti H. Range uncertainties in proton therapy and the role of Monte Carlo simulations. Phys Med Biol. 2012;57:R99–117. 22571913 10.1088/0031-9155/57/11/R99 PMC3374500

[10] Agostinelli S Allison J Amako K Apostolakis J Zschiesche D. Geant4 – a simulation toolkit. Nucl Instrum Methods Phys Res Sect A. 2003;506:250.

[11] Paganetti H Niemierko A Ancukiewicz M. Relative biological effectiveness (RBE) values for proton beam therapy. Int J Radiat Oncol Biol Phys. 2002;53:407–21. 12023146 10.1016/s0360-3016(02)02754-2

[12] Wilson RR. Radiological use of fast protons. Radiology. 1946;47:487–91. 20274616 10.1148/47.5.487

[13] Lawrence JH Tobias CA Born JL. Pituitary irradiation with high-energy proton beams: a preliminary report. Cancer Res. 1958;18:121–34. 13511365

[14] Cooper ID. Bibliometrics basics. J Med Libr Assoc. 2015;103:217–8. 26512226 10.3163/1536-5050.103.4.013 PMC4613387

[15] Chen C Song M. Visualizing a field of research: a methodology of systematic scientometric reviews. PLoS One. 2019;14:e0223994. 31671124 10.1371/journal.pone.0223994 PMC6822756

[16] Smith A Gillin M Bues M. The M. D. Anderson proton therapy system. Med Phys. 2009;36:4068–83. 19810479 10.1118/1.3187229

[17] Mohan R Grosshans D. Proton therapy – present and future. Adv Drug Deliv Rev. 2017;109:26–44. 27919760 10.1016/j.addr.2016.11.006 PMC5303653

[18] Yap J De Franco A Sheehy S. Future developments in charged particle therapy: improving beam delivery for efficiency and efficacy. Front Oncol. 2021;11:780025. 34956897 10.3389/fonc.2021.780025 PMC8697351

[19] Moreno AC Frank SJ Garden AS. Intensity modulated proton therapy (IMPT) – The future of IMRT for head and neck cancer. Oral Oncol. 2019;88:66–74. 30616799 10.1016/j.oraloncology.2018.11.015 PMC6615027

[20] Mohan R Das IJ Ling CC. Empowering intensity modulated proton therapy through physics and technology: an overview. Int J Radiat Oncol Biol Phys. 2017;99:304–16. 28871980 10.1016/j.ijrobp.2017.05.005 PMC5651132

[21] Kooy HM Grassberger C. Intensity modulated proton therapy. Br J Radiol. 2015;88:20150195. 26084352 10.1259/bjr.20150195 PMC4628542

[22] Chang JY Zhang X Knopf A. Consensus guidelines for implementing pencil-beam scanning proton therapy for thoracic malignancies on behalf of the PTCOG thoracic and lymphoma subcommittee. Int J Radiat Oncol Biol Phys. 2017;99:41–50. 28816159 10.1016/j.ijrobp.2017.05.014

[23] Schuemann J Dowdell S Grassberger C Min CH Paganetti H. Site-specific range uncertainties caused by dose calculation algorithms for proton therapy. Phys Med Biol. 2014;59:4007–31. 24990623 10.1088/0031-9155/59/15/4007 PMC4136435

[24] Schuemann J Giantsoudi D Grassberger C Moteabbed M Min CH Paganetti H. Assessing the clinical impact of approximations in analytical dose calculations for proton therapy. Int J Radiat Oncol Biol Phys. 2015;92:1157–64. 26025779 10.1016/j.ijrobp.2015.04.006 PMC4509834

[25] Paganetti H Jiang H Parodi K Slopsema R Engelsman M. Clinical implementation of full Monte Carlo dose calculation in proton beam therapy. Phys Med Biol. 2008;53:4825–53. 18701772 10.1088/0031-9155/53/17/023

[26] Yepes PP Eley JG Liu A. Validation of a track repeating algorithm for intensity modulated proton therapy: clinical cases study. Phys Med Biol. 2016;61:2633–45. 26961764 10.1088/0031-9155/61/7/2633

[27] Kohno R Hotta K Nishioka S Matsubara K Tansho R Suzuki T. Clinical implementation of a GPU-based simplified Monte Carlo method for a treatment planning system of proton beam therapy. Phys Med Biol. 2011;56:N287–294. 22036894 10.1088/0031-9155/56/22/N03

[28] Giantsoudi D Schuemann J Jia X Dowdell S Jiang S Paganetti H. Validation of a GPU-based Monte Carlo code (gPMC) for proton radiation therapy: clinical cases study. Phys Med Biol. 2015;60:2257–69. 25715661 10.1088/0031-9155/60/6/2257 PMC7788741

[29] Paganetti H. Relative biological effectiveness (RBE) values for proton beam therapy. Variations as a function of biological endpoint, dose, and linear energy transfer. Phys Med Biol. 2014;59:R419–472. 25361443 10.1088/0031-9155/59/22/R419

[30] Lühr A von Neubeck C Pawelke J.; “Radiobiology of Proton Therapy”. “Radiobiology of Proton Therapy”: results of an international expert workshop. Radiother Oncol. 2018;128:56–67. 29861141 10.1016/j.radonc.2018.05.018

[31] Paganetti H Blakely E Carabe-Fernandez A. Report of the AAPM TG-256 on the relative biological effectiveness of proton beams in radiation therapy. Med Phys. 2019;46:e53–78. 30661238 10.1002/mp.13390 PMC9559855

[32] Xiao Y Qiu M Huang W. Global status of research on radiotherapy for rectal cancer: a bibliometric and visual analysis. Front Public Health. 2022;10:962256. 36003628 10.3389/fpubh.2022.962256 PMC9393343

[33] Combs SE. Does proton therapy have a future in CNS tumors? Curr Treat Options Neurol. 2017;19:12. 28365895 10.1007/s11940-017-0447-4

[34] Patriarca A Fouillade C Auger M. Experimental set-up for FLASH proton irradiation of small animals using a clinical system. Int J Radiat Oncol Biol Phys. 2018;102:619–26. 30017793 10.1016/j.ijrobp.2018.06.403

[35] Zhang Q Cascio E Li C. FLASH investigations using protons: design of delivery system, preclinical setup and confirmation of FLASH effect with protons in animal systems. Radiat Res. 2020;194:656–64. 32991708 10.1667/RADE-20-00068.1

[36] Cunningham S McCauley S Vairamani K. FLASH proton pencil beam scanning irradiation minimizes radiation-induced leg contracture and skin toxicity in mice. Cancers (Basel). 2021;13:1012. 33804336 10.3390/cancers13051012 PMC7957631

[37] Buonanno M Grilj V Brenner DJ. Biological effects in normal cells exposed to FLASH dose rate protons. Radiother Oncol. 2019;139:51–5. 30850209 10.1016/j.radonc.2019.02.009 PMC6728238

[38] Diffenderfer ES Verginadis II Kim MM. Design, implementation, and in vivo validation of a novel proton FLASH radiation therapy system. Int J Radiat Oncol Biol Phys. 2020;106:440–8. 31928642 10.1016/j.ijrobp.2019.10.049 PMC7325740

[39] Ogata T Teshima T Kagawa K. Particle irradiation suppresses metastatic potential of cancer cells. Cancer Res. 2005;65:113–20. 15665286

[40] Gameiro SR Malamas AS Bernstein MB. Tumor cells surviving exposure to proton or photon radiation share a common immunogenic modulation signature, rendering them more sensitive to T cell-mediated killing. Int J Radiat Oncol Biol Phys. 2016;95:120–30. 27084634 10.1016/j.ijrobp.2016.02.022 PMC4834148

[41] Su CW Hou MM Huang PW. Proton beam radiotherapy combined with anti-PD1/PDL1 immune checkpoint inhibitors for advanced hepatocellular carcinoma. Am J Cancer Res. 2022;12:1606–20. 35530291 PMC9077059

[42] Kim H Park S Jung HA. Phase II trial of combined durvalumab plus tremelimumab with proton therapy for recurrent or metastatic head and neck squamous cell carcinoma. Cancer Res Treat. 2023;55:1104–12. 37202212 10.4143/crt.2023.502 PMC10582547

