# Supplementary material for: A review and bibliometric analysis of global research on proton radiotherapy
Source: Medicine (Baltimore). 2024 May 10;103(19):e38089. doi: 10.1097/MD.0000000000038089 (PMC11081588; doi:10.1097/MD.0000000000038089)
Supplement: Supplementary file 1 [file medi-103-e38089-s002.docx]

**Supplementary Table S1.** Top 10 cocited references related to PBT research.

| **Rank** | **Article Titles** | **Author** | **NOC** |
| --- | --- | --- | --- |
| 1 | Range uncertainties in proton therapy and the role of Monte Carlo simulations | Paganetti H | 543 |
| 2 | GEANT4-a simulation toolkit | Agostinelli S | 532 |
| 3 | Relative biological effectiveness (RBE) values for proton beam therapy | Paganetti H | 324 |
| 4 | Relative biological effectiveness (RBE) values for proton beam therapy. Variations as a function of biological endpoint, dose, and linear energy transfer | Paganetti H | 300 |
| 5 | The 200-MeV proton therapy project at the Paul Scherrer Institute: Conceptual design and practical realization | Pedroni E | 281 |
| 6 | TOPAS: An innovative proton Monte Carlo platform for research and clinical applications | Perl J | 276 |
| 7 | Intensity modulation methods for proton radiotherapy | Lomax A | 273 |
| 8 | The calibration of CT Hounsfield units for radiotherapy treatment planning | Schneider U | 251 |
| 9 | Radiological use of fast protons | Wilson RR | 249 |
| 10 | Intensity modulated proton therapy and its sensitivity to treatment uncertainties 2: the potential effects of inter-fraction and inter-field motions | Lomax AJ | 231 |

NOC, number of citations.
